# NMR, RP-HPLC-PDA-ESI-MS^n^, and RP-HPLC-FD Characterization of Green and Oolong Teas (*Camellia sinensis* L.)

**DOI:** 10.3390/molecules26175125

**Published:** 2021-08-24

**Authors:** Anatoly P. Sobolev, Arianna Di Lorenzo, Simone Circi, Cristina Santarcangelo, Cinzia Ingallina, Maria Daglia, Luisa Mannina

**Affiliations:** 1 Institute for Biological Systems, Magnetic Resonance Laboratory “Segre-Capitani”, CNR, Via Salaria Km 29.300, 00015 Monterotondo, Italy; anatoly.sobolev@cnr.it; 2 Dipartimento di Scienze del Farmaco, Università degli Studi di Pavia, Viale Taramelli 12, 27100 Pavia, Italy; arianna.dilorenzo01@universitadipavia.it; 3 Department of Chemistry and Technologies of Drugs, Laboratory of Food Chemistry, Sapienza University of Rome, P.le Aldo Moro 5, 00185 Rome, Italy; scirci89@gmail.com (S.C.); luisa.mannina@uniroma1.it (L.M.); 4 Department of Pharmacy, University of Naples Federico II, 80138 Naples, Italy; cristina.santarcangelo@unina.it; 5 International Research Center for Food Nutrition and Safety, Jiangsu University, Zhenjiang 212013, China

**Keywords:** green and oolong teas, bioactive metabolites, targeted and untargeted analyses

## Abstract

Untargeted (NMR) and targeted (RP-HPLC-PDA-ESI-MS^n^, RP-HPLC-FD) analytical methodologies were used to determine the bioactive components of 19 tea samples, characterized by different production processes (common tea and GABA tea), degrees of fermentation (green and oolong teas), and harvesting season (autumn and spring). The combination of NMR data and a multivariate statistical approach led to a statistical model able to discriminate between GABA and non-GABA teas and green and oolong teas. Targeted analyses showed that green and GABA green teas had similar polyphenol and caffeine contents, but the GABA level was higher in GABA green teas than in regular green tea samples. GABA oolong teas showed lower contents of polyphenols, caffeine, and amino acids, and a higher content of GABA, in comparison with non-GABA oolong teas. In conclusion, the results of this study suggest that the healthy properties of teas, especially GABA teas, have to be evaluated via comprehensive metabolic profiling rather than only the GABA content.

## 1. Introduction

The chemical composition of tea has been extensively studied, leading to the identification of many phytochemicals responsible for its healthy properties [1,2,3,4,5]. These bioactive compounds can be subdivided into polyphenolic components and non-polyphenolic compounds. Green tea leaves are particularly rich in polyphenols (up to 30% of the dry weight), mainly flavan-3-ols, with EGCG being the most represented (9–13%) [6,7]. In oolong and black teas, the polyphenol concentration is lower and depends on the degree of fermentation. In fact, during the oxidation process, flavan-3-ols undergo oxidation and polymerization reactions, catalyzed by polyphenol oxidase and peroxidase, leading to the formation of theaflavins (TFs) and thearubigins (TRs), the most representative compounds of oolong and black teas, respectively [6,8]. The chemical structure of the major TFs is known, but TRs are still under investigation, due to their high molecular weight and complexity [9]. Other flavonoids occurring in tea leaves include flavonol glycosides, such as quercetin, kaempferol, and myricetin mono-, di-, and triglycosides linked to pentose (xylose or arabinose) or hexose (glucose, galactose, rhamnose). Acylated flavonols (particularly p-coumaroyl derivatives) and methylated flavonols have also been detected by means of sophisticated hyphenated techniques [10]. Non-flavonoid polyphenols such as benzoic acids (i.e., gallic acid and galloylquinic acid) and hydroxycinnamic acid derivatives (i.e., caffeoylquinic acid and *p*-coumaroylquinic acid) are also present in tea leaves. Among the non-polyphenolic components, xanthines and proteic and non-proteic amino acids are the most representative categories. Tea beverages contain caffeine and, in lower amounts, theobromine and theophylline [11]. The total free amino acid content usually accounts for 1–4% of the dry weight of tea leaves, and the types of free amino acids and their proportion in tea are closely related to tea aroma and taste [12,13]. Theanine, the most abundant amino acid occurring in tea (37% of the whole amino acid content) and glutamate, responsible for the umami taste, are important healthy tea compounds [14,15].

Over recent years, the growing demand for tea characterized by a high concentration of healthy compounds led to the development of different analytical approaches towards the study of tea beverages [16]. Among the studies found in the literature, Gotti et al. (2009) studied the differentiation of green tea samples by the analysis of catechins via CD-MEKC [17]; meanwhile, Meng et al. (2017) used proton NMR spectroscopy and near-infrared spectroscopy to identify the geographic origin of oolong tea [18]. In the same year, a comparative study was performed on the volatile components present in fresh leaves of four Dianhong tea cultivars, using chromatography coupled with mass spectrometry, multivariate data analysis, and descriptive sensory analysis [19]. Fare clic o toccare qui per immettere il testo. Nevertheless, important issues about tea chemistry still need to be studied further. Indeed, despite all the advances in tea chemical characterization, as described above, in a recent review, Engelhardt pointed out that there is still much to be conducted in terms of definition and authenticity [16]. In particular, a combined targeted and untargeted analytical approach has to be applied to overcome each technique’s limits, in order to obtain more information and a more complete picture.

Thus, the current study was designed to explore how different variables such as degrees of fermentation (green and oolong), harvesting season (autumn and spring), and production process (GABA and non-GABA) affect the chemical profile of tea through the application of a multi-methodological analytical protocol.

This analytical approach, previously used for the study of different food matrices [20,21,22], consists of three analytical techniques, untargeted NMR spectroscopy and targeted RP-HPLC-PDA-ESI-MS^n^ and RP-HPLC-FD.

## 2. Results and Discussion

### 2.1. Untargeted NMR Analysis

**Figure 1:**
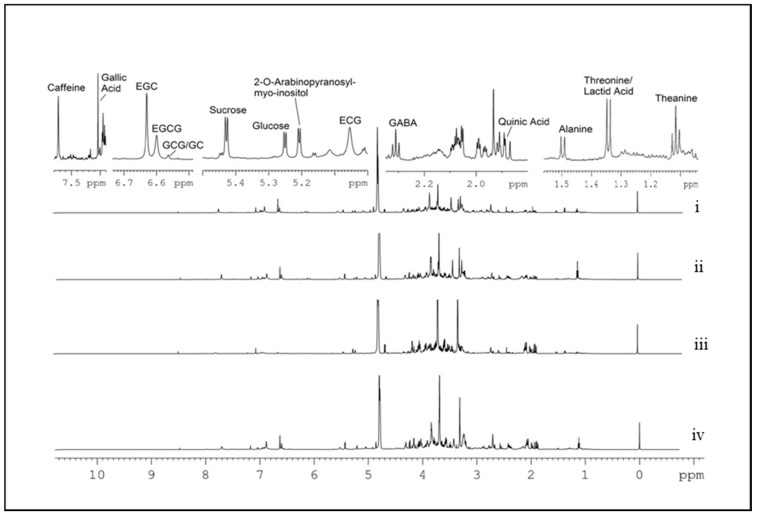
^1^H spectra of the four types of teas. The fourteen ^1^H NMR signals selected for the statistical analysis are indicated at the top. i = GABA green tea GGT1; ii = green tea GT2; iii = GABA oolong tea GOT3; iv = oolong tea OTA1.

**Figure 2:**
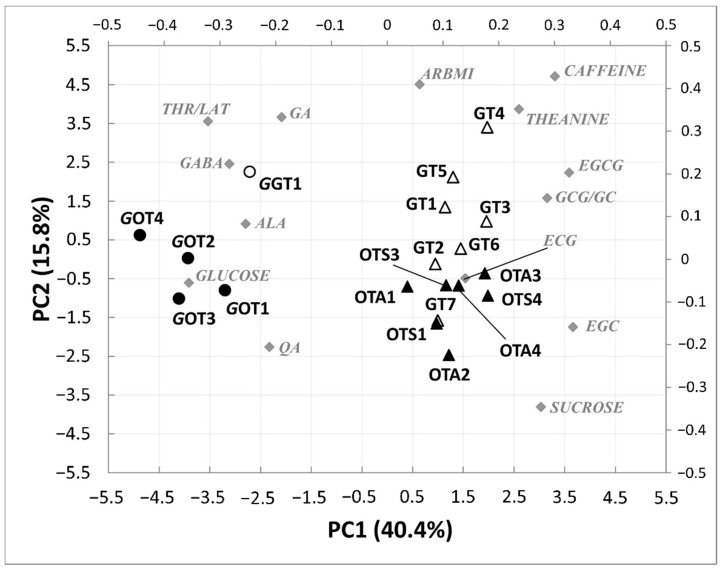
Biplot diagram of PCA performed using 14 resonances measured in the ^1^H NMR spectra of 19 tea samples belonging to four different types of tea (unfilled triangles = green teas; unfilled circles = GABA green teas; filled triangles = oolong teas; filled circles = GABA oolong teas).

Nineteen tea samples belonging to four types of tea were submitted to untargeted NMR analysis (Table 1). The ^1^H spectra (Figure 1) of the analyzed teas were assigned according to the literature [22], with the presence of theanine at 1.118 ppm, threonine/lactate (THR/LAT) at 1.348 ppm, alanine (ALA) at 1.498 ppm, quinic acid (QA) at 1.888 ppm, gamma aminobutyric acid (GABA) at 2.314 ppm, epicatechin gallate (ECG) at 5.057 ppm, 2-O-arabinopyranosyl-myo-inositol (ARBMI) at 5.207 ppm, α-glucose at 5.255 ppm, sucrose at 5.427 ppm, GCG/GC at 6.563 ppm, EGCG at 6.597 ppm, EGC at 6.631 ppm, GA at 7.037 ppm, and caffeine at 7.709 ppm. In the investigated samples, the concentration of these metabolites turned out to be different and can contribute towards grouping samples according to their commercial denomination. Therefore, an explorative analysis of the entire dataset was undertaken using PCA to highlight dissimilarities and similarities. Figure 2 shows a PCA sample biplot (PC2 versus PC1) with the first two PCs accounting for 56.2% of the variability within the data. PC1 provided differentiation between GABA and non-GABA teas, whereas PC2 was mostly responsible for discriminating oolong from green teas. The loadings reported in the biplot (Figure 2) clearly show the variables responsible for the separation of GABA and non-GABA teas. Comparisons of metabolites from different tea groups suggest that GABA teas (loadings lying on the left side of the PC1 axis) contained a high level of GABA, glucose, ALA, THR/LAT, and QA, whereas non-GABA teas (both oolong and green teas) showed higher caffeine, theanine, EGC, EGCG, GCG/GC, and sucrose contents (loadings lying on the right side of the PC1 axis). Therefore, all the fine homogenate dried GABA tea leaves were found to have very low concentrations of green tea characteristic polyphenolic compounds. Moreover, the GT7 sample was found to be similar to oolong teas being characterized by a low content of ARBMI, theanine, and caffeine. Although the literature data reported a significant effect of pedoclimatic conditions (including climate condition, soil) on teas’ chemical composition [23,24,25], independently of the production season (autumn or spring), green teas were characterized by very high levels of caffeine, ARBMI, and theanine, which provided a great stimulating capacity, whereas oolong tea samples showed a higher level of sucrose. These results are in agreement with Lee et al., Le Gall et al., and Tarachiwin et al., by which caffeine, gallic acid, and theanine are believed to be possible quality markers of green tea, and thus their high contents are responsible for the high quality of the tea [26,27,28].

### 2.2. RP-HPLC-PDA-ESI-MS^n^ Analysis

**Figure 3:**
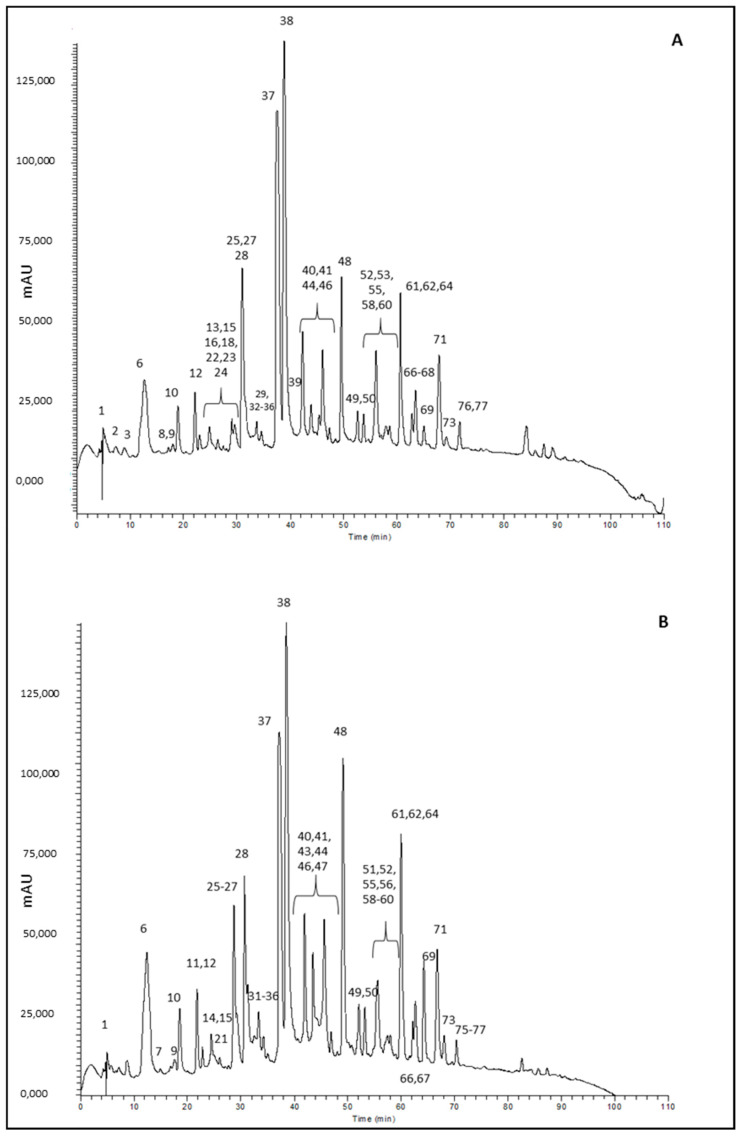
Chromatograms, acquired as total scan PDA, of (**A**) OTS3 and (**B**) GT1 tea extracts at 5 mg/mL.

Tea beverages were submitted to RP-HPLC-PDA-ESI-MS^n^ analysis to investigate their metabolite profile. RP-HPLC-PDA-ESI-MS^n^ (Figure 3) allowed the identification of 90 compounds, as reported in Tables S1–S3. Identification was accomplished by comparing experimental data (retention time, MS and MSn spectra) with those available in the literature, and with commercial standard compounds where possible.

Caffeine and theobromine identified in peaks 12 and 37, respectively, are common xanthine alkaloids occurring in tea leaves [29]. Fare clic o toccare qui per immettere il testo. QA (peak 1 in Table S1) and galloylquinic acid (peak 6 in Table S1) were detected in all tea beverages (Tables S2 and S3).

Indeed, oolong teas are a great GA source, as GA (peak 19 in Table S1) was detected in all GOTs [30]. In *G*OT4, the gallic acid methyl ester (peak 20 in Table S1) was also detected. This RP-HPLC-PDA-ESI-MS^n^ method allowed the identification of hydroxycinnamic acids, among which there were three isoforms of *p*-coumaroylquinic acid (peaks 32, 41, and 44 in Table S1), that differ thanks to the MS/MS fragment ion ratio, as shown by Clifford et al. [31]. Moreover, both 3-caffeoylquinic acid and 5-caffeoylquinic acid were detected in a few green tea samples (i.e., GT1, GT5, GT6, and GT7 in Table S2).

As far as flavones (Table S1) are concerned, six glycosilated apigenins were identified on the basis of the MS/MS spectra and the retention time [32]. Apigenin 6-C-glucosyl-8-C-arabinoside (peak 49 in Table S1) and 6-C-glucosyl-8-C-arabynoil apigenin (peak 50 in Table S1) were detected in all teas, as reported by Dou et al. [33] (Tables S2 and S3). 6,8-C-di-pentosyl apigenin (peak 62) was identified in all teas, with the exception of *G*OT1, *G*OT2, *G*OT3, and OTA2 (Tables S2 and S3). Apigenin hexosides (apigenin glucoside and apigenin galactoside), di-hexosides (peaks 46 and 59 Table S1), and rhamnosyl-hexoside (peak 58 Table S1) were detected, but not in all the analyzed tea samples (Tables S2 and S3). In detail, the GT6 tea showed the presence of two compounds with these MS/MS spectra and different retention times [32], revealing the presence of both apigenin hexosides and di-hexosides. All teas showed the presence of apigenin rhamnosyl-hexoside (peak 58 Table S1), with the exception of GT5, GT6, and GT7 (Table S2).

In addition, 27 tannins (Table S1) were identified: 6 hydrolyzable tannins, and 21 proanthocyanidins (Table S1), assigned by comparing retention times and MS/MS spectra with the literature data [34]. Of the hydrolyzable tannins, strictinin (peak 24 Table S1), galloylglucose (peak 4 Table S1), and digalloylglucose (peak 21) were identified in all samples, with the exception of GT1 and OTA2, GT1 and *G*OT4, and *G*OT1, OTA2, OTA4, OTS3, and OTS4, respectively (Tables S2 and S3). Trigalloylglucose (peak 43 Table S1) was detected only in green teas, with the exception of GT5 and GT7 (Tables S2 and S3). Regarding proanthocyanidins, procyanidin (peaks 15, 25, and 34, Table S1), prodelphinidin (peak 7, Table S1), and procyanidin gallate (peaks 35 and 36, Table S1) were detected in the 19 tea beverages (Tables S2 and S3). Prodelphinidin gallate (peak 11, Table S1) was not detected in *G*OT1 and *G*OT2 and in all oolong teas.

Among flavan-3-ols (Table S1), two isoforms of (epi)catechin-(epi)gallocatechin (peaks 8 and 14 in Table S1) and (epi)afzelechingallate-(epi)catechingallate (peaks 18 and 23 in Table S1) were identified in all teas, with the exception of *G*GT1, *G*OT1, and *G*OT2, and GT1, GT4, and *G*GT1, respectively. Interestingly, among the oolong teas, (epi)gallocatechin-(epi)catechingallate (peak 26 in Table S1) was identified only in autumn-harvested teas (i.e., OTA1, OTA2, OTA3, OTA4), suggesting the potential use of this compound as a marker if this finding is confirmed in a larger number of samples. The presence of (epi)catechin-(epi)gallocatechingallate, whose parent ion and fragmentation were reported by Lui et al. [35], was not registered in GGT1, GOT1, and GOT2.

Following a previously published description of theasinensins performed by Dai et al. [36] and by Li et al. [37], studying MS/MS fragmentations both in GABA oolong and non-GABA oolong teas, three theasinensins (peaks 2, 5, 13, 16, 22, 29, and 39, Table S1) were identified (theasinensin A, theasinensin B, and theasinensin C).

Considering flavonols (Table S1), 2 myricetin derivatives, 13 quercetin derivatives, and 12 kaempferol derivatives were identified. Myricetin hexoside (peak 53) was identified in all tea beverages, with the exception of GT1. Myricetin hexosyl-rutinoside (peak 51) was detected only in GT1, GT5, and GT7 (Table S2). Quercetin rutinoside (peak 67) was identified in all teas. In the GT6 and GT7 teas, another peak (no. 80 in Table S1) was registered with the same *m/z* but different MS/MS spectra: the presence of a major fragment ion at *m/z* 463 suggested the loss of a rhamnosyl moiety (−146 Da), leading to identification as a mono-glycosilated quercetin [13]. This fragmentation led to the consideration that the two sugars could be linked at different positions; thus, the proposed structure for peak 80 (Table S1) is quercetin 3-hexosyl-7-rhamnoside. Two isomers of quercetin, characterized by the same UV–Vis and MS/MS spectra but with different retention times, corresponding to quercetin hexose conjugates (quercetin glucoside and quercetin galactoside), were detected in all teas, with the exception of GT5 and GT7. Finally, quercetin rhamnosyl derivatives were identified in GT4 (dirhamnosyl derivative) and GT1, GT3, GT4, and GT5 (rhamnosyl-hexosyl-rhamnoside). Many kaempferol derivatives have already been detected in all types of tea (Table S1) [32]. Our analysis showed the presence of kaempferol hexoside (peak 76, Table S1) and kaempferol hexosyl-rutinoside (peak 77, Table S1) in all tea beverages, whilst kaempferol rutinoside was present in all teas, with the exception of *G*GT1 (Table S2). Kaempferol rhamnosyl derivatives were also identified in GT4 (rhamnosyl and dirhamnosyl derivatives), GT7 (rhamnosyl derivative), and GT1, GT2, GT3, GT4, and *G*GT1 (kaempferol rhamnosyl-hexosyl-rhamnoside). The *G*OT4 and OTA3 teas were characterized by the presence of kaempferol pentoside, which was not identified in the corresponding spring-harvested tea, OTS3 (Tables S2 and S3). All peaks were assigned according to the description provided by Del Rio et al. [9]. Fare clic o toccare qui per immettere il testo. Due to the common loss of 146 amu between the pseudomolecular ion and the principal fragment ion, a peculiar shift in absorbance spectra toward 310–316 nm (due to the addition of hydroxyl-aromatic organic acids [9,32,38] Fare clic o toccare qui per immettere il testo.), and high retention times (peaks 79, 82, 83, 84, 89, and 90 in Table S1), these compounds were identified as *p*-coumaroyl conjugates of quercetin and kaempferol. For peaks 63 and 70 (Table S1), which showed the same features as the peaks reported above, the shorter retention time did not support this assignment. Thus, they were identified as tetra-glycosilated quercetin and tetra-glycosilated kaempferol, respectively.

Concerning flavan-3-ols, GC and EGCG were detected in all the tea beverages analyzed (Tables S2 and S3). For the two monomers, catechin and its epimer EC, the former was identified in all green teas, with the exception of the GT5 tea, but only in a few oolong teas (i.e., *G*OT4, OTA3, OTS1, OTS3, and OTS4). EGC and EGCG were identified in all teas, with the exception of GT6 and *G*GT1 and *G*OT3, respectively. Catechingallate (peak 47, Table S1) was detected only in GT1 and GT4 (Table S2). Gallocatechingallate was identified in a larger number of samples, including some oolong teas. Two methylated flavan-3-ols were identified both in green and in oolong teas: epigallocatechin-3-O-(3-O methyl)-gallate and epicatechin-3-O-(3-O methyl)-gallate, with the latter detected in a larger number of samples [38]. (Epi)afzelechin, a less common flavan-3-ol, was also detected in some samples, together with its derivative, methoxyepiafzelechin gallate.

Finally, some catechin condensation products, formed during the fermentative process, were identified. In *G*OTs, the main TFs (i.e., theaflavin, theaflavin-gallate, and theaflavin-digallate) were identified, resulting from the dimerization of a catechin and a gallocatechin and responsible for the bright and red-orange appearance of oolong tea [19]. Their absence in oolong teas could be attributed to their low degree of fermentation.

In conclusion, RP-HPLC-PDA-ESI-MS^n^ analysis revealed some differences in the metabolite profiles. Xathine alkaloids (i.e., caffeine and theobromine), quinic acid and galloylquinic acid, some flavones (i.e., apigenin 6-C-glucosyl-8-C- and 6-C-glucosyl-8-C-arabynoil apigenin), condensed tannins (i.e., procyanidin, prodelphinidin, and procyanidin gallate), flavonols (i.e., quercetin rutinoside, kaempferol hexoside, and kaempferol hexosyl-rutinoside), and flavan-3-ols (i.e., gallocatechin and (-)-epigallocatechin-3-gallate) were found in all tea samples. No relevant differences were found between green teas and GABA green teas, but the three Japanese teas (GT5, GT6, and GT7) are the only ones in which caffeoylquinic acids were detected. Among oolong teas, also in this case, regarding theasinensins, no relevant differences were found between non-GABA oolong teas and GABA oolong teas. Moreover, the autumn-harvested tea Anxi Ti Kuan Yin differs from the other tea samples in its chemical composition because of the absence of *p*-coumaroyl quinic acids and strictinin, the presence of tetraglycosilated kaempferol, and fewer flavones than in the other oolong teas. Further research on a larger number of oolong tea samples should be carried out to verify whether (epi)gallocatechin-(epi)catechingallate can be considered a marker of autumn-harvested teas.

### 2.3. Quantification of Targeted Bioactive Molecules by RP-HPLC-PDA

**Figure 4:**
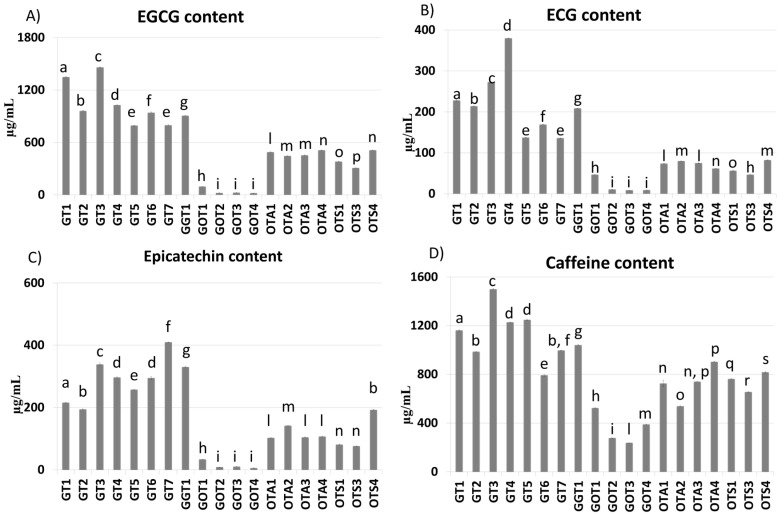
EGCG (**A**), ECG (**B**), EC (**C**), and caffeine (**D**) concentrations (μg/mL of tea beverage) in the 19 analyzed teas. Data are expressed as mean of three independent measurements ± SD; different letters indicate statistically significant differences (*p* < 0.05) between two groups.

The GA, caffeine, EGCG, ECG, EC, and catechin contents are reported in Figure 4. As expected, EGCG was the most abundant flavan-3-ol in all the analyzed teas, and its content was higher in green teas than in oolong teas (Figure 4A).

Among oolong teas, GABA oolong teas held the lowest content of all quantified catechins (Figure 4B–D).

A possible explanation for these results is that non-GABA oolong teas are characterized by a low degree of fermentation during which flavan-3-ols do not undergo polymerization reactions, remaining in their original form. The two shaded Japanese green teas (GT5 and GT7) resulted in being less rich in catechins, with the exception of the EC content (Figure 4C), partially confirming the results obtained by Zheng et al., according to which tea leaves accumulate catechins to protect themselves from UV-B radiation damage [39].

Our results indicate that Mao Feng tea is the richest green tea beverage in terms of EGCG. At the operating conditions, catechin was found only in a few samples, among which were two oolong tea beverages, OTS1 and OTS3, which showed a catechin content of 164.83 ± 2.13 μg/mL and 144.48 ± 2.14 μg/mL, respectively, and a GABA green tea, *G*GT1, which showed a content of 361.93 ± 3.91 μg/mL.

In regard to caffeine, our results are in agreement with the data previously published by Horzic et al. (2009) showing that green teas contained more caffeine than oolong teas, with the only exception of oolong tea OTA4, which was found to be richer than green tea GT6 [40]. The caffeine concentration in the analyzed green teas generally ranged from 0.9 to 1.2 mg/mL, with the exception of the GT3 and GT6 teas, which showed a concentration of 1.5 mg/mL and 0.79 mg/mL, respectively (Figure 4D).

### 2.4. RP-HPLC-FD Analysis

**Figure 5:**
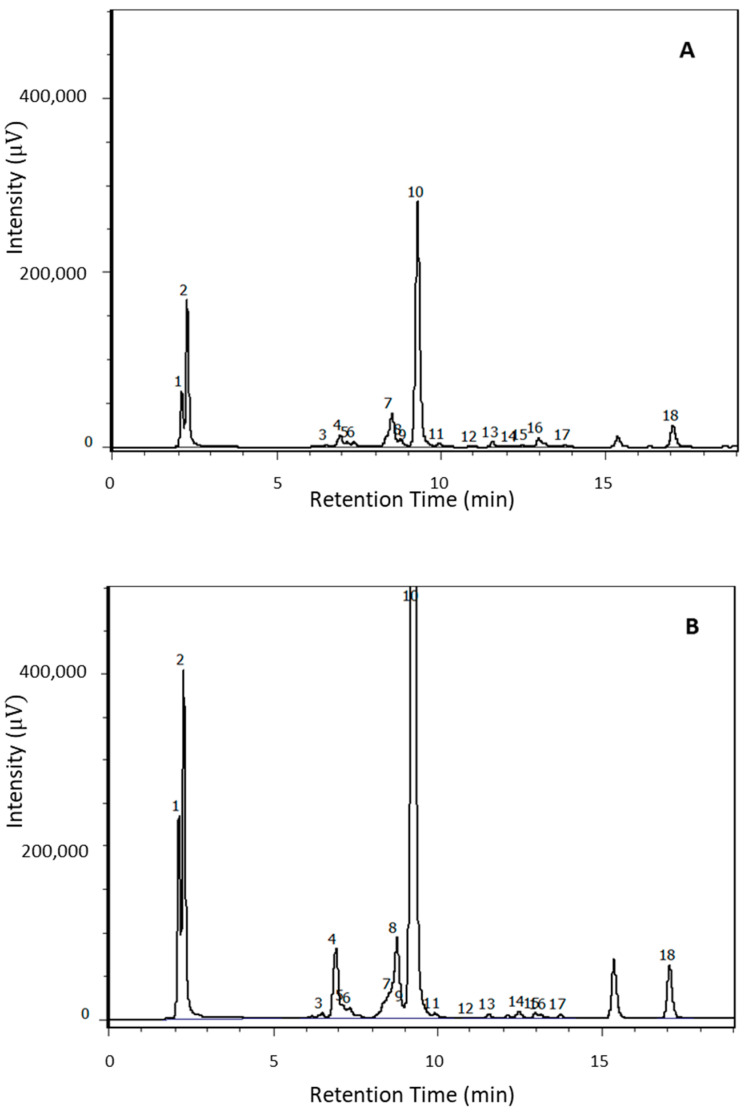
(**A**) HPLC/FLD chromatograms of OTA2 tea extract at the concentration of 0.5 mg/mL. (**B**) HPLC/FLD chromatograms of GT5 tea extract at the concentration of 0.5 mg/mL. The analysis showed the presence of (1) aspartic acid; (2) glutamic acid; (3) serine; (4) glutamine; (5) glycine; (6) threonine; (7) alanine; (8) arginine; (9) GABA 1; (10) theanine; (11) tyrosine; (12) cysteine; (13) valine; (14) GABA 2; (15) phenylalanine; (16) isoleucine; (17) leucine; (18) proline.

Free amino acid identification was based on the chromatographic behaviors of their derivatives, compared with those of derivatized commercial standards. A total of 18 proteic and non-proteic amino acids were identified (Figure 5).

Among these compounds, we quantified those that exert the most important healthy properties and thus could be considered markers of high-quality teas used as a functional beverage (i.e., GABA, glutamic acid, glutamine, and theanine).

**Figure 6:**
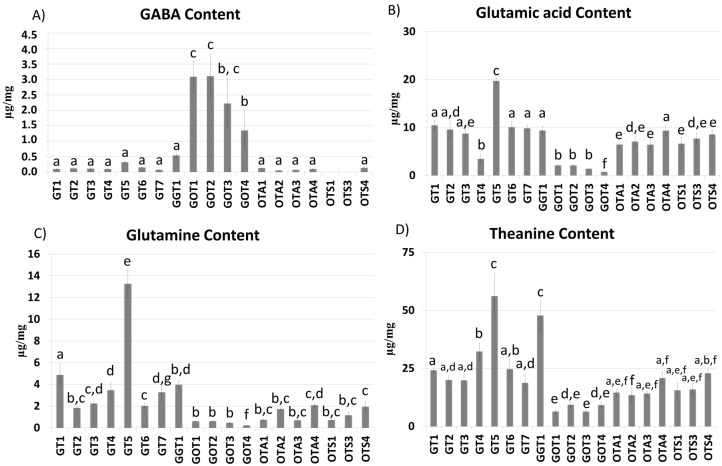
GABA (**A**), glutamic acid (**B**), glutamine (**C**), and theanine (**D**) concentrations (μg/mg of tea dried residue) in the 19 analyzed teas. Data are expressed as mean of three independent measurements ±SD; different letters indicate statistically significant differences (*p* < 0.05) between two groups.

Although the concentration was extremely variable, the results (Figure 6) show that, according to Yu et al. [41], on average, theanine (with a concentration ranging from 6.38 to 56.15 μg/mg) and its precursor, glutamic acid (with a concentration ranging from 0.75 to 19.71 μg/mg), were the most abundant amino acids present in the tea samples. GT5 differed from the other green teas, having the highest concentrations of glutamic acid, glutamine, and theanine. A possible explanation of the high level of glutamic acid, a proteinaceous amino acid, could be that in this tea sample, high proteolysis occurs, probably due to the effects of preharvest treatments such as dark treatment of tea plants [42]. The high content of glutamic acid could also justify the high content of glutamine and theanine derived from glutamic acid by reaction with free ammonia and ethylamine, catalyzed by L-glutamine synthetase and L-theanine synthetase, respectively [43]. The high content of theanine in GT5 is in agreement with Lee et al., who demonstrated that green tea grown in shady conditions presented a high theanine content [26]. No relevant differences were registered in terms of the amino acid content among oolong teas and, in particular, between the same oolong teas from the spring and the autumn harvest, leading to the hypothesis that the harvest season does not seem to influence the amino acid content.

As far as GABA is concerned (Figure 6A), it is known that GABA is produced in response to stress, including oxygen deficiency and nitrogen treatment used to obtain this type of tea [44]. With the exception of the GGT1 beverage, which showed a GABA content similar to that found for non-GABA tea samples, as expected, the GABA concentration was higher in GABA tea samples, allowing for discriminating between GABA teas and non-GABA teas.

With regard to the glutamic acid content, the data represented in Figure 6B show that GABA tea beverages presented the lowest amounts of glutamic acid, confirming its conversion into GABA during the manufacturing process [45]. Moreover, apart from GT5 mentioned above, GT4, which showed a significantly lower concentration of glutamic acid in comparison with the other green tea samples, and OTA4, which presented a higher glutamic acid content similar to that found in green tea samples, on average, showed a glutamic acid concentration higher than that determined for the oolong tea samples. This result could be explained by literature data showing that leaf cell disruption during the tea fermentation process occurring in the preparation of oolong teas resulted in protein degradation to form free proteinaceous amino acids, which decreased quickly as they were metabolized to aromatic compounds [41].

In terms of glutamine (Figure 6C), GABA tea samples showed a low glutamine content, with the exception of the GGT1 beverage, which showed a glutamine content similar to that found for non-GABA tea samples. Probably, the low glutamine concentration found in GABA tea samples was due to the fact that glutamic acid is used in the synthesis of GABA and not in that of glutamine. Comparing green and oolong teas, no relevant differences were registered, even though oolong teas presented a lower mean glutamine concentration than green teas.

Finally, regarding the theanine content (Figure 6D), once again, the GT5 tea beverage resulted in being the richest one. Although the green tea GT7 was also shade-grown, it did not have a theanine content comparable with tea GT5. Comparing green and oolong teas, no statistically significant differences were registered in the theanine content, with the exception of GT4. This result appears to disagree with that obtained by Guo et al. in 2018, who demonstrated that in oolong teas, the theanine content is very low due to the fact that this compound contributes to the formation of 2, 5-dimethyl-pyrazine, a key roasted peanutty flavor present in oolong tea [46].

## 3. Materials and Methods

### 3.1. Reagents and Materials

Tea samples were purchased from an Italian specialist tea shop (La Teiera Eclettica, Milan, Italy). The selected teas differ in the degree of fermentation (green and oolong teas) and in the production process (GABA teas and non-GABA teas) (Table 1). Samples GT5, GT6, and GT7 were submitted to different light exposures: GT6 was unshaded, and GT5 and GT7 were shaded for 10 and 20 days, respectively.

EC, ECG, EGCG, and L-theanine were purchased from PhytoLab GmbH & Co. KG (Vestenbergsgreuth, Germany). GA, (±)-catechin, caffeine, glutamic acid, glutamine, GABA, sodium acetate, triethylamine, tetrahydrofurane, ortho-phthalaldehyde, fluorenylmethylchloroformate, Na_2_CO_3_, HPLC-MS-grade methanol, HPLC-grade acetonitrile, and formic acid solution 1 M were obtained from Sigma Aldrich Chemical Company (St. Louis, MO, USA). HPLC-grade water was obtained from an LC-PakTM Millex system (Millipore Coorporation, Billerica, MA). D_2_O and TSP were purchased from Euriso-top (Saint-Aubin, France). D_2_O phosphate buffer for NMR samples (400 mM, pH 7.00) was prepared using potassium phosphate monobasic and potassium phosphate dibasic from Sigma Aldrich.

### 3.2. Sample Preparation for NMR analysis

Preparation of tea infusions for NMR analysis required a fine homogenization with a mortar and pestle of dried tea leaves to obtain a better recovery of soluble minor metabolites with respect to conventional infusions. Dried leaves from each tea sample were finely ground to obtain a homogeneous matrix. Deuterated water (1 mL) was added to 50 mg of each ground tea sample in a 1.5 mL Eppendorf tube. The mixture was introduced to a water bath at 85 °C for 4 min, followed by cooling to room temperature and filtering through cotton wool. The limpid solution (400 μL) and the D_2_O phosphate buffer (300 μL, 400 mM, pH 7.00, with a small quantity of EDTA and 1 mM of TSP as an internal standard) were mixed and transferred to a 5 mm NMR tube.

### 3.3. NMR Analysis

NMR spectra were recorded at 27 °C on a Bruker AVANCE 600 spectrometer (Milan, Italy) operating at a proton frequency of 600.13 MHz and equipped with a Bruker multinuclear z-gradient inverse probe head. The ^1^H spectra of tea samples were acquired by co-adding 400 transients with a recycle delay of 7.1 s, using a 90° pulse of 13 μs and 32 K data points. The water signal was suppressed using solvent presaturation during the relaxation delay. Data processing was carried out with Bruker TOPSPIN 3.5 software. An exponential function with a line broadening factor of 0.3 Hz was applied, the spectra were manually phased, and polynomial baseline correction was applied. All selected NMR peaks were integrated manually with the same integral limits for a given peak in all spectra. The integrals of the selected resonances were normalized by setting the integral of TSP resonance at 0.00 ppm to 100.

### 3.4. Sample Preparation for Spectrophotometric and HPLC Analyses

Tea extracts were prepared by infusion to mimic the conditions commonly used for the preparation of tea beverages, as reported by Di Lorenzo et al. [14]. Briefly, 25 g of each tea in 500 mL of mineral water with its composition and content is reported in Table S4. The timing of infusion and temperature were set as suggested by the supplier (Table 1). After infusion, the suspension was cooled at room temperature for 10 min, and the supernatant was filtrated through a paper filter under vacuum. The extracts were subdivided into different aliquots and freeze dried to be submitted to qualitative and quantitative HPLC analyses.

### 3.5. RP-HPLC-PDA-ESI-MS^n^ Analysis

RP-HPLC-PDA-ESI-MS^n^ analysis was performed using a Thermo Finnigan Surveyor Plus HPLC, equipped with a quaternary pump, a Surveyor UV−Vis diode array detector, and an LCQ Advantage Max ion trap mass spectrometer (Thermo Fisher Scientific, Waltham, MA, USA), connected through an ESI source. Separation was achieved on a Zorbax Eclipse XDB-C18 column (150 mm × 4.6 mm, 5 μm), equipped with a Hypersil gold C18 (10 mm × 2.1 mm, 5 μm) precolumn, both from Agilent (Waldbronn, Germany). The mobile phase consisted of water acidified with 0.1% formic acid (eluent A) and methanol (eluent B) and was eluted in a gradient as follows: from 10 to 70% B in 84 min, from 70 to 80% B in 5 min, from 80 to 100% B in 10 min, followed by a 5 min isocratic run of 100% B. Total run time was 105 min, including column reconditioning. The flow rate was maintained at 0.3 mL/min, and the autosampler and the column temperatures were maintained at 4 and 25 °C, respectively. Tea extracts were analyzed at the concentration of 5 mg/mL in water, and 5 μL of the solution was injected into the chromatographic system. Chromatograms were registered at 210, 254, and 280 nm; spectral data were collected within the range of 200−800 nm for all peaks.

HPLC-ESI-MS^n^ data were acquired under positive and negative ionization modes, using the Xcalibur software. The ion trap operated in full scan (100–2000 *m/z*), data-dependent scan, and MS^n^ modes. To obtain MS^2^ data, a 35% collision energy and an isolation width of 2 *m/z* were applied. To optimize MS operating conditions, a preliminary experiment was performed: 5 μg/mL caffeine (0.1% formic acid and methanol, 50:50, %*v*/*v*) and 10 μg/mL (±)-catechin (0.1% formic acid and methanol, 50:50, %*v*/*v*) solutions were directly infused through the ESI interface at a flow rate of 25 μL/min into the mass spectrometer. The optimized conditions were as follows: sheath gas 60, capillary temperature 220 °C, spray voltage 4.5, auxiliary gas 25 and 20, capillary voltage −47.20 V and 5 V, for the negative and the positive ionization mode, respectively.

### 3.6. RP-HPLC-PDA Analysis

The quantification of caffeine and flavan-3-ols in tea samples was performed through an RP-HPLC-PDA method developed and validated according to Marchese et al. (2014) [22] Fare clic o toccare qui per immettere il testo., on a 1100 Agilent HPLC system (Agilent, Waldbronn, Germany), equipped with a gradient quaternary pump and a diode array detector. The Agilent Chemstation software was used for HPLC system control and data processing. Separation was achieved using the experimental conditions reported above. Calibration curves were prepared with a mixture of standards in a range of concentration between 20 and 2000 μg/mL with five concentration levels. Each analysis was performed in triplicate (R^2^ = 0.997). For each dry extract, 5 mg was weighed and solubilized in 1 mL of water. Before analysis, the samples were filtrated on PES membrane 0.22 μm (MiniSart, Sartorius, USA).

### 3.7. Determination of Glutamic acid, Glutamine, γ-Amino Butyric Acid, and Theanine

Before amino acid analysis, the tea samples were hydrolyzed with 6M HCl in glass sterilized tubes at 120 °C for 24 h under nitrogen atmosphere. Ortho-phthalaldeyde (OPA) and fluorenylmethylchloroformate (FMOC) were used in free amino acid derivatization to obtain derivatives from primary amino acids and secondary amino acids. Derivatization was achieved according to the Jasco autosampler program previously reported by Di Lorenzo et al. [14]. Amino acid analysis was performed with the Jasko X-LC system provided with a 3159AS autosampler, 3185PU Xtreme high pressure pumps, a 3080DG degasser, a CO2060 Plus column oven compartment, and a 3020FP fluorescence detector connected to an HP ProDesk G1 400 MT processor, Intel Core i5. Separation was carried out with a Hypersil ODS (250 mm × 2.1 mm, 5 μm) column, and the mobile phase was formed by 20 mM sodium acetate solution with 0.018% *v*/*v* triethylamine (TEA) and 0.3% *v*/*v* tetrahydrofuran (THF), at pH 7.2 (eluent A), and 100 mM sodium acetate buffer with 35% methanol and 45% acetonitrile, at pH 7.2 (eluent B). The temperature of the column was set at 45 °C, with injection of 1 μL and a run time of 17 min. The fluorometric detector was set at emission λ = 456 nm – excitation λ = 342 for OPA derivatives, and emission λ = 272 nm – excitation λ = 312 nm for FMOC derivatives. ChromNav software was used for processing the data. GABA, glutamic acid, glutamine, and theanine were selected as external standards. Calibration curves were prepared with six concentration levels in a range of concentration between 80 and 2 μg/mL. Each analysis was performed in triplicate (R^2^ = 0.998).

### 3.8. Statistical Analysis

Statistical analysis was carried out with Statistical Package for the Social Sciences (IBM SPSS 21.0 for Windows). Results were expressed as means ± SD, and *p* < 0.05 was considered statistically significant. The statistical significance of the data was assessed through one-way ANOVA. Where significant differences were found, Tukey’s post hoc test was used to determine the differences between the groups involved.

NMR data were submitted to the Statistica software package for Windows. PCA was carried out. The data were pre-processed before statistical analysis; the variables were mean-centered, and each variable was divided by its standard deviation (autoscaling).

## 4. Conclusions

The chemical profiles of different processed and fermented teas were interpreted based on the results of this study. This work may improve authenticity issues of tea matrices, and the combined targeted and untargeted analytical approach here applied serves as a guide for a more comprehensive metabolic profiling. Indeed, the healthy properties of teas have to be considered not only based on a single component but also on the entire chemical profile, given that many other components may account for the healthy properties, such as polyphenols, xanthines, and amino acids. Further studies need to be carried out on larger tea samples to finally build a tea database.

## Figures and Tables

**Table 1:** List of selected teas, their abbreviations, the degree of fermentation, the origin, the infusion conditions as suggested by the supplier, and the dry residue obtained after freeze drying.

N.	Tea Sample	Abbreviation	Degree of Fermentation	Country of Origin	Infusion Parameter	Dry Residue (mg/50 mg)
1	Lung Ching	GT1 ^1^	Green	China	85 °C 3 min	12.3
2	Gunpowder	GT2	Green	Zhejiang Province (China)	85 °C 3 min	10.5
3	Mao Feng	GT3	Green	Anhui Province (China)	85 °C 4 min	17.0
4	Jade Snow	GT4	Green	Guangxi region (China)	80 °C 4 min	11.1
5	Kabusecha	GT5	Green	Japan	70 °C 2 min	13.7
6	Sencha	GT6	Green	Japan	80 °C 2 min	12.7
7	Gyokuro Kyushu	GT7	Green	Japan	75 °C 2 min	13.9
8	GABA green	*G*GT1 ^2^	Green	Japan	80 °C 2 min	11.2
9	Dong Ding	OTA1 ^3^	Low degree of fermentation	Taiwan	85 °C 4 min	7.9
10	Anxi Ti Kuan Yin	OTA2	Low degree of fermentation	Fujian region (China)	85 °C 4 min	8.2
11	Si Ji Chun	OTA3	Low degree of fermentation	Taiwan	85 °C 4 min	8.0
12	Wenshan Baozhong	OTA4	Low degree of fermentation	Taiwan	85 °C 4 min	8.5
13	Dong Ding	OTS1 ^4^	Low degree of fermentation	Taiwan	85 °C 4 min	7.1
14	Si Ji Chun	OTS3	Low degree of fermentation	Taiwan	85 °C 4 min	6.6
15	Wenshan Baozhong	OTS4	Low degree of fermentation	Taiwan	85 °C 4 min	11.7
16	GABA oolong	*G*OT1 ^5^	Low degree of fermentation	Taiwan	80 °C 2 min	7.2
17	GABA oolong	*G*OT2	Low degree of fermentation	Taiwan	80 °C 2 min	3.6
18	GABA oolong	*G*OT3	Low degree of fermentation	Taiwan	80 °C 2 min	4.2
19	GABA oolong	*G*OT4	Low degree of fermentation	Taiwan	80 °C 2 min	2.2

^1^ GT = green tea. ^2^ *G*GT = GABA green tea. ^3^ OTA = oolong tea. ^4^ GOT = GABA oolong tea. ^5^ GOT = GABA oolong tea.

## Data Availability

The data presented in this study are available on request from the corresponding author.

## Supplementary Materials

The following are available online. Table S1: Chromatographic behavior (retention time, RT), UV, MS, and MS/MS data of the compounds identified in the 19 analyzed tea samples, Table S2: Presence (+) or absence (−) of the identified secondary metabolites in the analyzed green tea samples, Table S3: Presence (+) or absence (−) of the identified secondary metabolites in the analyzed oolong teas, Table S4: Composition of water utilized for tea infusion, expressed as mg of anion or cation dissolved in 1 L of water.

## Sample Availability

Samples of the compounds are available from the authors.

## Abbreviations

6-aminoquinolyl-N-hydroxysuccinimidyl carbamate (ACQ), catechin (C), catechin gallate (CG), epicatechin (EC), electrochemical detector (ECD), epicatechin-3-gallate (ECG), epigallocatechin (EGC), (−)-epigallocatechin-3-gallate (EGCG), fluorometric detector (FD), 9-fluorenylmethyloxycarbonyl chloride (FMOC-Cl), γ-amino butyric acid (GABA), gallic acid (GA), gallic acid equivalents (GAE), gallocatechin (GC), gallocatechin-3-gallate (GCG), green tea (GT), GABA green tea (GGT), GABA oolong tea (GOT), high-resolution mass spectrometry (HR-MS), N-acetyl-cysteine (NAC), ortho-phthalaldehyde (OPA), oolong tea from autumn harvest (OTA), oolong tea from spring harvest (OTS), photodiode array detector (PAD), principal component analysis (PCA), reverse-phase (RP) HPLC, theaflavins (TFs), total polyphenol content (TPC), thearubigins (TRs), 3-(trimethylsilyl)-propionic-2,2,3,3-d4 acid sodium salt (TSP), CD-MEKC (cyclodextrin-mediated micellar electrokinetic chromatography), NMR (nuclear magnetic resonance).
