# Supplementary material for: NMR, RP-HPLC-PDA-ESI-MSn, and RP-HPLC-FD Characterization of Green and Oolong Teas (Camellia sinensis L.)
Source: Molecules. 2021 Aug 24;26(17):5125. doi: 10.3390/molecules26175125 (PMC8434197; doi:10.3390/molecules26175125)
Supplement: Supplementary file 1 [file molecules-26-05125-s001.zip › molecules-1298596-supplementary.pdf]

Supplementary

Table S1. Chromatographic behavior (retention time, RT), UV, MS and MS/MS data of the compounds identified in the 19 analysed tea samples.

Peak	RT (min)	ë max (nm)	*m/z [M-H]⁻*	HPLC-ESI-MS/MS *m/z* (% of base peak)	Proposed structure
**Xanthines**					
12	22.5	222, 273	181[a]	137(100)	theobromine
37	38.7	230, 270	195[a]	138(100)	caffeine
**Organic and phenolic acids**					
1	5.2	211	191	127(100), 85(80)	quinic acid
6	13.6	219, 274	343	191(100), 169(10), 125(2)	galloylquinic acid
17	26.6	225, 280, 306	353	191(100), 179(65), 135(30)	3-caffeoylquinic acid
19	27.5	219, 270	169	125(100)	gallic acid

20	28.3	221, 273	183	168(100), 139(90), 124(15)	gallic acid methylester
31	33.8	228, 280, 306	353	191 (100), 179 (3)	5-caffeoylquinic acid
32	34.1	211, 330	337	163(100), 191(10), 119(10), 173(5)	3-*p*-coumaroylquinic acid
41	43.5	211, 310	337	191(100), 173(25), 163(10)	5-*p*-coumaroylquinic acid
44	46.3	211, 309	337	173(100), 163(15), 191(10)	4-*p*-coumaroylquinic acid
**Flavones**					
46	47.7	210, 270, 333	593	473(100), 353(25), 383(15), 503(30)	dihexosyl-apigenin
49	52.9	210, 275	563	503(70), 473(100), 443(80), 383(45), 353(50)	Apigenin 6-*C*-glucosyl-8-*C*-arabinoside
50	54.2	210, 275	563	503(30), 473(100), 443 (100), 383(40), 353(40)	Apigenin 6-*C*-glucosyl-8-*C*-arabinoside
55	56.5	220, 268, 331	431	311(100), 341(5)	hexosyl apigenin
58	58.6	211, 258, 352	577	413(100), 293(30), 457(10), 353(2)	rhamnosyl-hexosyl apigenin
59	59.08	211, 271, 336	593	413(100), 293(10), 473(10)	dihexosyl-apigenin

60	59.6	220, 268, 331	431	311(100), 341(40)	hexosyl apigenin
62	60.6	256, 353	533	443(100), 473(50), 353(20)	6,8-*C*-dipentoside apigenin
64	61.6	211, 258, 352	577	413(100), 293(30), 457(10), 353(2)	rhamnosyl-hexosyl apigenin
**Flavan-3-ols**					
10	19.5	220, 270	305	261(40), 221(85), 219(75), 179(100), 165(35), 125(20)	gallocatechin
27	31.3	240, 270	305	261(40), 179(100), 219(70), 165(35) 221(85), 125(25)	epigallocatechi n
28	32.4	211, 278	289	245(100), 205(40), 179(15), 203(40), 109(5)	catechin
38	39.2	233, 274	457	331(70), 169(100), 305(35)	epigallocatechi n gallate
40	43.1	232, 278	289	245(100), 205(40), 109(5), 137(10), 125(2)	epicatechin
42	44.6	233, 274	457	331(70), 169(100), 305(35)	gallocatechinga llate
45	46.4	211, 276	471	183(100), 305(35), 161(10)	(epi)gallocatech in 3-*O*-(3-*O*-methyl) gallate

47	49.6	230, 276	441	289(100), 331(20), 169(25)	catechingallate
48	52.8	230, 276	441	289(100), 331(20), 169(25)	epicatechin-gallate
52	55.8	219, 270, 352	455	289(100), 183(25)	epicatechin-3-*O*-(3-methyl)-gallate
54	56.2	209, 277	455	289(100), 183(35), 245(5)	methoxyepiafzelechin-gallate
57	57.2	209, 270	425	273(100), 169(40), 125(5)	epiafzelechin-gallate
**Theaflavins**					
85	84.8	223, 270, 364, 470	867	715(65), 697(100), 679(15), 527(45), 545(25), 559(20)	theaflavin-digallate
86	84.9	213, 269, 367, 478	715	527(100), 545(80), 563(70), 501(40), 407(10)	theaflavin-gallate
87	85.8	215, 270, 364	563	545(100), 527(30), 519(55), 501(30), 407(70), 379(50)	theaflavin
88	86.6	213, 269, 367, 478	715	527(30), 545(60), 563(100), 501(20), 407(60)	theaflavin-gallate
**Flavonols**					
51	54.5	210, 273	787	316(100), 769(30), 359(20), 625(15), 725(20)	myricetin hexosyl rutinoside

53	55.9	215, 266, 350	479	316(100), 317(30)	myricetin hexoside
56	56.9	225, 269, 349	755	609(100), 447(20), 301(10)	quercetin rhamnosyl-hexosyl-rhamnoside
61	60.4	211, 256, 356	771	301(100), 609(10), 463(2)	quercetin hexosyl rutinoside
63	61.5	226, 269, 342	917	771(100), 753(2), 300(2)	quercetin-tetraglycoside
65	61.6	226, 264, 349	593	447(100), 431(25), 301(15)	quercetin dirhamnoside
66	62.7	207, 267, 356	463	301(100)	quercetin hexoside
67	63.1	211, 256, 357	609	301(100), 271(15)	quercetin rutinoside
68	63.4	207, 267, 356	463	301(100)	quercetin hexoside
70	65.2	228, 267, 339	901	755(100), 431(20), 284(2)	kaempferol-tetraglycoside
74	69.8	214, 264, 348	447	301(100), 300(30)	quercetin rhamnoside
69	64.5	210, 267, 348	755	285(100)	kaempferol hexosylrutinoside

71	67.3	210, 267, 348	755	285(100)	kaempferol hexosylrutinoside
72	68.1	230, 264, 344	577	431(100), 285(20)	kaempferol dirhamnoside
73	68.5	206, 268	447	285(40), 284(100), 327(20)	kaempferol hexoside
75	70.2	201, 270	739	285(100), 257(10), 593(10)	kaempferol rhamnosyl-hexosyl-rhamnoside
76	70.7	206, 268	447	285(40), 284(100), 327(20)	kaempferol hexoside
77	70.7	207, 266, 345	593	285(100)	kaempferol rutinoside
78	74.6	215, 268, 315	417	284(100), 285(20)	kaempferol pentoside
79	75.9	228, 270, 320	901	285(40), 755(100)	kaempferol *p*-coumaroyl-rhamnosyl-hexoside
80	77.6	226, 270, 330	609	463(100), 301 (20)	quercetin 3-hexosyl-7-rhamnoside
81	78.4	226, 264, 344	431	285(100), 284(40)	kaempferol rhamnoside
82	82.1	259, 315	1079	933 (100), 915 (20), 301 (2)	quercetin-3-*O*-hexosylrhamnosyl-(*p*-coumaroyl-

					hexosyl)-hexoside
83	83.3	224, 269, 316	917	771(100), 753(2), 300(2)	quercetin-3-*O*-*p*-coumaroyl-hexosyl-rhamnsosyl-hexoside
84	84.8	269, 316	901	755 (100)	quercetin-3-*O*-coumaroyl-pentosyl-rhamnosyl hexoside
89	90.0	267, 314	901	755 (100)	quercetin-3-*O*-coumaroyl-pentosyl-rhamnosyl hexoside
90	96.3	220, 270	885	739(100), 431(20), 285(10)	kaempferol-3-*O*-*p*-coumaroyl-dirhamnosyl hexoside
**Tannins**					
2	7.71	216, 267	609	471(100), 591(80), 565(20), 525(30)	theasinensin C
3	9.6	210, 260	331	169(100), 271(80), 211(40), 193(20), 125(15)	galloylglucose
4	12.0	210, 260	331	169(100), 271(80), 211(40), 193(20), 125(15)	galloylglucose
5	12.1	216, 260	609	565(100), 591(90), 471(60), 525(20)	theasinensin C

7	15.2	211	609	483(30), 441(100), 423(70), 305(20), 591(29)	prodelphinidin
8	17.9	220, 280	593	423(100), 467(85), 575(60), 305(40), 289(30)	(epi)catechin-(epi)gallocatechin
9	18.1	211	609	483(30), 441(100), 423(70), 305(20), 591(29)	prodelphinidin
11	22.0	211, 275	761	609(100), 423(80), 305(20), 591(70)	prodelphinidin gallate
13	22.8	217, 270	761	609(40), 591(100), 453(10)	theasinensin B
14	24.2	220, 280	593	423(100), 467(85), 575(60), 305(40), 289(30)	(epi)catechin-(epi)gallocatechin
15	24.4	211, 278	577	425(100), 407(40), 289(10), 451(25)	procyanidin
16	25.9	216, 274	761	609(100), 591(95), 453(70)	theasinensin B
18	26.8	210, 254	865	739(15), 695(100), 577(45)	(epi)afzelechingallate-(epi)catechingallate
21	28.7	221, 275	483	271(100), 331(20), 169(10)	digalloylglucose
22	28.8	216, 276	761	609(50), 591(100), 453(10)	theasinensin B

23	29.4	210, 254	865	739(15), 695(100), 577(45)	(epi)afzelechingallate-(epi)catechingallate
24	29.5	226, 274	633	301(100), 463(15)	strictinin
25	29.9	211, 278	577	425(100), 407(40), 289(10), 451(25)	procyanidin
26	30.3	202, 280	745	593(100), 423(60), 575(50), 727(15), 243(5)	(epi)gallocatechin-(epi)catechingallate
29	32.5	223, 271	913	743(100), 761(50), 591(80), 573(45)	theasinensin C
30	33.8	221, 275	483	271(100), 331(20), 169(10)	digalloylglucose
33	34.2	211, 276	745	559(65), 407(100), 619(90), 577(65), 441(40)	(epi)catechin-(epi)gallocatechingallate
39	41.9	223, 271	913	743(100), 761(50), 591(80), 573(45)	theasinensin C
34	35.1	211, 278	577	425(100), 407(40), 289(10), 451(25)	procyanidin
35	35.9	211, 277	729	559(100), 577(95), 407(20), 441(5) 603(25), 451(35), 711(15), 289(10)	procyanidin gallate
36	38.4	211, 277	729	407(80), 577(80), 711(20), 559(100),	procyanidin gallate

				451(50), 603(50), 441(50), 289(10)	
43	45.8	220, 280	635	465(100), 483(70), 313(20)	trigalloylglucose

a) compounds revealed in the positive ionization mode

Table S2. Presence (+) or absence (-) of the identified secondary metabolites in the analysed green tea samples.

Compound	GT1	GT2	GT3	GT4	GT5	GT6	GT7	GGT
**Xanthines**								
theobromine	+	+	+	+	+	+	+	+
caffeine	+	+	+	+	+	+	+	+
**Organic and phenolic acids**								
quinic acid	+	+	+	+	+	+	+	+
gallic acid	-	-	-	-	-	-	-	-
gallic acid methyl ester	-	-	-	-	-	-	-	-
galloylquinic acid	+	+	+	+	+	+	+	+
3-*p*-coumaroylquinic acid	+	+	+	+	+	-	+	+
4-*p*-coumaroylquinic acid	+	+	+	+	+	+	+	+
5-*p*-coumaroylquinic acid	+	+	+	+	+	-	-	+
5-caffeoylquinic acid	+	-	-	-	+	+	+	-
3-caffeoylquinic acid	-	-	-	-	+	-	-	-
**Flavones**								
di-hexosyl apigenin	+ 2 isomers	+ 2 isomers	+ 2 isomers	+	+ 2 isomers	+ 2 isomers	+	+ 2 isomers

Apigenin 6-*C*-glucosyl-8-*C*-arabinoside	+	+	+	+	+	+	+	+
Apigenin 6-*C*-glucosyl-8-*C*-arabinoside	+	+	+	+	+	+	+	+
di-pentosyl apigenin	+	+	+	+	+	+	+	+
apigenin hexoside	+	+	+	+	+	+ 2 isomers	+	-
apigenin rhamnosyl-hexoside	+ 2 isomers	+	+	+	-	-	-	+
**Flavan-3-ols**								
gallocatechin	+	+	+	+	+	+	+	+
epigallocatechin	+	+	+	+	+	-	+	-
catechin	+	+	+	+	-	+	+	+
epicatechin	+	+	+	+	+	+	+	+
catechin gallate	+	-	-	+	-	-	-	-
epicatechin gallate	+	+	+	+	+	+	+	+
epigallocatechin gallate	+	+	+	+	+	+	+	+
gallocatechin gallate	+	+	+	+	-	-	+	+
(epi)gallocatechin-3-*O*-(3-*O* methyl)-gallate	-	-	-	-	-	-	-	-
(epi)catechin-3-*O*-3-*O*-methylgallate	+	+	+	-	-	-	-	-
(epi)afzelechin	-	-	-	-	-	+	+	+
methoxyepiafezelechin gallate	-	-	-	-	-	-	-	-
**Theaflavins**								
theaflavin	-	-	-	-	-	-	-	-
theaflavin gallate	-	-	-	-	-	-	-	-
theaflavin digallate	-	-	-	-	-	-	-	-
**Tannins**								
galloylglucose	-	+	+	+	+	+	+	+
digalloylglucose	+	+ 2 isomers	+ 2 isomers	+ 2 isomers	+	+	+	+
trigalloylglucose	+	+	+	+	-	+	-	+
strictinin	-	+	+	+	+	+	+	+
procyanidin	+ 3 isomers	+ 3 isomers	+ 3 isomers	+ 3 isomers	+ 3 isomers	+ 3 isomers	+ 3 isomers	+ 2 isomers
procyanidin gallate	+ 2 isomers	+ 2 isomers	+ 2 isomers	+ 2 isomers	+ 2 isomers	+ 2 isomers	+	+

								2 isomers
prodelphinidin	+ 2 isomers	+ 2 isomers	+ 2 isomers	+	+ 2 isomers	+ 2 isomers	+ 2 isomers	+
prodelphinidin gallate	+	+	+	+	+	+	+	+
(epi)catechin-(epi)gallocatechin	+	+	+	+ 2 isomers	+	+	+ 2 isomers	-
(epi)afzelechin gallate-(epi)catechin gallate	-	+	+	-	+ 2 isomers	+ 2 isomers	+	-
(epi)gallocatechin-(epi)catechin gallate	+	+	+	+	-	-	-	-
(epi)catechin-(epi)gallocatechin gallate	+	+	+	+	+	+	+	-
theasinensin A	-	-	-	-	-	-	-	-
theasinensin B	-	-	-	-	-	-	-	-
theasinensin C	-	-	-	-	-	-	-	-
**Flavonols**								
myricetin hexoside	-	+	+	+	+	+	+	+
myricetin hexosyl rutinoside	+	-	-	-	+	-	+	+
quercetin rhamnoside	-	-	-	+	-	-	-	-
quercetin dirhamnoside	-	-	-	+	-	-	-	-
quercetin hexoside	+	+ 2 isomers	+ 2 isomers	+ 2 isomers	-	+	-	+
quercetin hexosyl rutinoside	+	+	-	+ 2 isomers	+	+	+	+
quercetin rutinoside	+	+	+	+	+	+ 2 isomers	+ 2 isomers	+
quercetin rhamnosyl-hexosyl-rhamnoside	+	-	+	+	+	-	-	-
quercetin tetraglycosilated	-	-	-	-	-	-	+ 2 isomers	-
quercetin-3-O-coumaroyl-pentosyl-rhamnsoyl-hexoside	-	-	-	-	-	-	-	-
quercetin-3-*O*-*p*-hexosylrhamnosyl-(*p*-coumaroylhexosyl)-hexoside	-	-	-	-	-	-	-	-

kaempferol hexoside	+ 2 isomers	+ 2 isomers	+ 2 isomers	+	+	+ 2 isomers	+	+
kaempferol pentoside	-	-	-	-	-	-	-	-
kaempferol rutinoside	+	+	+	+	+	+	+ 2 isomers	-
kaempferol hexosyl-rutinoside	+ 2 isomers	+ 2 isomers	+ 2 isomers	+	+ 2 isomers	+ 2 isomers	+	+ 2 isomer s
kaempferol rhamnosyl-hexosyl-rhamnoside	+ 2 isomers	+	+ 2 isomers	+ 2 isomers	-	-	-	-
kaempferol rhamnoside	-	-	-	+	-	-	+	-
kaempferol dirhamnoside	-	-	-	+	-	-	-	-
kaempferol tetraglycosilated	-	-	-	-	-	-	-	-
kaempferol p-coumaroyl-hexoside	-	-	-	-	+	-	-	-
kaempferol-3-O-p-coumaroyldirhamn soylhexoside	-	-	-	-	-	-	-	-

Table S3. Presence (+) or absence (-) of the identified secondary metabolites in the analysed oolong teas.

Compound	OTA1	OTA2	OTA3	OTA4	OTS1	OTS3	OTS4	GOT1	GOT2	GOT3	GOT4
**Xanthines**											
theobromine	+	+	+	+	+	+	+	+	+	+	+
caffeine	+	+	+	+	+	+	+	+	+	+	+
**Organic and phenolic acids**											
quinic acid	+	+	+	+	+	+	+	+	+	+	+
gallic acid	-	-	-	-	-	-	-	+	+	+	+
gallic acid methyl ester	-	-	-	-	-	-	-	-	-	-	+
galloylquinic acid	+	+	+	+	+	+	+	+	+	+	+
3-*p*-coumaroilquinic acid	+	-	+	+	+	+	+	+	+	+	+
4-*p*-coumaroilquinic acid	+	-	+	+	+	+	+	+	+	+	+
5-*p*-coumaroilquinic acid	+	-	+	+	+	+	+	+	+	+	+
5-caffeoylquinic acid	-	-	-	-	-	-	-	-	-	-	-
3-caffeoylquinic acid	-	-	-	-	-	-	-	-	-	-	-
**Flavones**											
di-hexosyl apigenin	+ 2 isomers	+ 2 isomers	+ 2 isomers	+ 2 isomers	+ 2 isomers	+	+ 2 isomers	+ 2 isomers	+ 2 isomers	+ 2 isomers	+ 2 isomers
Apigenin 6-C-glucosyl-8-C-arabinoside	+	+	+	+	+	+	+	+	+	+	+
Apigenin 6-C-glucosyl-8-C-arabinoside	+	+	+	+	+	+	+	+	+	+	+
di-pentosyl apigenin	+	-	+	+	+	+	+	-	-	-	+
apigenin hexoside	+	-	+	+	+	+	+	-	-	+	+
apigenin rhamnosyl-hexoside	+	+ 2 isomers	+	+ 2 isomers	+	+ 2 isomers	+ 2 isomers	+	+	+ 2 isomers	+ 2 isomers
**Flavan-3-ols**											
gallocatechin	+	+	+	+	+	+	+	+	+	+	+
epigallocatechin	+	+	+	+	+	+	+	+	+	+	+
catechin	-	-	+	-	+	+	+	-	-	-	+
epicatechin	+	+	+	+	+	+	+	+	+	+	+

catechin gallate	-	-	-	-	-	-	-	-	-	-	-
epicatechin gallate	+	+	+	+	+	+	+	+	+	-	+
epigallocatechin gallate	+	+	+	+	+	+	+	+	+	+	+
gallocatechin gallate	-	-	+	+	-	-	-	-	-	-	+
(epi)gallocatechin-3-*O*-(3-*O* methyl)-gallate	-	-	+	-	-	-	-	-	-	-	-
(epi)catechin-3-*O*-3-*O*-methylgallate	+	+ 2 isomers	+ 2 isomers	+ 2 isomers	+ 2 isomers	+ 2 isomers	+ 2 isomers	+	+	+ 2 isomers	+ 2 isomers
(epi)afzelechin	-	-	-	-	-	-	+	+	+	+	-
methoxyepiafezelechin gallate	-	-	-	-	-	-	-	-	-	-	-
**Theaflavins**											
theaflavin	-	-	-	-	-	-	-	+	+	+	+
theaflavin gallate	-	-	-	-	-	-	-	+ 2 isomers	+ 2 isomers	+ 2 isomers	+
theaflavin digallate	-	-	-	-	-	-	-	+	+	+	+
**Tannins**											
galloylglucose	+	+	+	+ 2 isomers	+	+	+	+	+	-	-
digalloylglucose	+	-	+ 2 isomers	-	+	-	-	-	+ 2 isomers	+ 2 isomers	-
trigalloylglucose	-	-	-	-	-	-	-	-	-	-	-
strictinin	+	-	+	+	+	+	+	+	+	+	+
procyanidin	+ 3 isomers	+ 2 isomers	+ 3 isomers	+ 2 isomers	+ 3 isomers	+ 3 isomers	+ 2 isomers	+ 3 isomers	+ 2 isomers	+ 3 isomers	+ 3 isomers
procyanidin gallate	+ 2 isomers	+ 2 isomers	+ 2 isomers	+ 2 isomers	+ 2 isomers	+ 2 isomers	+ 2 isomers	+ 2 isomers	+ 2 isomers	+ 2 isomers	+ 2 isomers
prodelphinidin	+	+	+ 2 isomers	+	+ 2 isomers	+	+ 2 isomers	+	+	+	+
prodelphinidin gallate	-	-	-	-	-	-	-	-	-	+	+
(epi)catechin-(epi)gallocatechin	+	+	+	+	+	+	+	-	-	+	+

(epi)afzelechin gallate-(epi)catechin gallate	+ 2 isomers	+	+	+ 2 isomers	+	+ 2 isomers	+	+	+	+ 2 isomers	+
(epi)gallocatechin-(epi)catechin gallate	+	+	+	+	-	-	-	-	-	-	-
(epi)catechin-(epi)gallocatechin gallate	+	+	-	+	+	+	+	+	+	+	+
theasinensin A	+	-	-	-	+	+	-	+	+	+	+ 2 isomers
theasinensin B	+ 2 isomers	+	+	+	+ 2 isomers	+ 2 isomers	+ 2 isomers	+	+	+	+ 3 isomers
theasinensin C	+	+	+	+	+	+	+	+	+ 2 isomers	+ 2 isomers	+ 2 isomers
**Flavonols**											
myricetin hexoside	+	+	+	+ 2 isomers	+	+	+	+	+ 2 isomers	+	+
myricetin hexosyl rutinoside	-	-	-	-	-	-	-	-	-	-	-
quercetin rhamnoside	-	-	-	-	-	-	-	-	-	-	-
quercetin dirhamnoside	-	-	-	-	-	-	-	-	-	-	-
quercetin hexoside	+ 2 isomers	+	+	+ 2 isomers	+ 2 isomers	+ 2 isomers	+ 2 isomers	+	+ 2 isomers	+ 2 isomers	+
quercetin hexosyl rutinoside	+	+	+	-	+	+	+	+	+	+	+
quercetin rutinoside	+	+	-	+	+	+	+	+	+	+	+
quercetin rhamnosyl-hexosyl-rhamnoside	-	-	-	-	-	-	-	-	-	-	-
quercetin tetraglycosilate	-	+ 2 isomers	+	+	+	-	-	-	+	+	+
quercetin-3-*O*-coumaroyl-pentosyl-rhamnsoyl-hexoside	-	+ 2 isomers	-	+	-	-	-	-	-	-	-

quercetin-3-*O*-p-hexosylrhamnosyl-(*p*-coumaroylhexosyl)-hexoside	-	-	-	+	-	-	-	-	-	-	-
kaempferol hexoside	+	+	+ 2 isomers	+ 2 isomers	+ 2 isomers	+ 2 isomers	+	+	+ 2 isomers	+ 2 isomers	+ 2 isomers
kaempferol pentoside	-	-	+	-	-	-	-	-	-	-	+
kaempferol rutinoside	+	+	+	+	+	+	+	+	+	+	+
kaempferol hexosyl-rutinoside	+ 2 isomers	+	+	+ 2 isomers	+ 2 isomers	+ 2 isomers	+ 2 isomers	+ 2 isomers	+ 2 isomers	+ 2 isomers	+ 2 isomers
kaempferol rhamnosyl-hexosyl-rhamnoside	-	-	-	-	-	-	-	-	-	-	-
kaempferol rhamnoside	-	-	-	-	-	-	-	-	-	-	-
kaempferol dirhamnoside	-	-	-	-	-	-	-	-	-	-	-
kaempferol tetraglycosilated	-	+	-	-	-	-	-	-	-	-	-
kaempferol *p*-coumaroyl-hexoside	-	-	-	-	-	-	-	-	-	-	-
kaempferol-3-*O*-p-coumaroyldirhamnsoylhexoside	-	+	-	-	-	-	+	+	+	+	-

Table S4. Composition of water utilized for tea infusion, expressed as mg of anion or cation dissolved in 1 L of water.

**Anions/Cations**	**mg/L**
HCO_3^-	7.5
F^-	< 0.1
NO_3^-	1.1
SO_4^{2-}	3.8
COA2$^+$	2.4
Na^+	1.7
Total dissolved solids	23.8
